# Therapeutic effect of mirror visual feedback therapy on neuropathic pain in patients with total brachial plexus injuries

**DOI:** 10.3389/fneur.2026.1875550

**Published:** 2026-08-05

**Authors:** Jingwei Wang, Zhuqing Huang, Yujie Lu, Ke Sha

**Affiliations:** 1 Department of Orthopaedics Trauma and Hand Surgery, First Affiliated Hospital of Guangxi Medical University, Nanning, Guangxi, China; 2 Department of Cardiothoratic Surgery Intensive Care Unit, Second Affiliated Hospital of Guangxi Medical University, Nanning, Guangxi, China; 3 Sichuan Cancer Hospital & Institute, Sichuan Cancer Center, School of Medicine, University of Electronic Science and Technology of China, Chengdu, Sichuan, China

**Keywords:** functional magnetic resonance imaging, mirror visual feedback therapy, neuropathic pain, sensorimotor cortex, total brachial plexus injury

## Abstract

**Objective:**

This study aimed to investigate the clinical efficacy of mirror visual feedback (MVF) therapy in treating neuropathic pain following total brachial plexus injury (TBPI), and to explore its effects on brain structure and functional activation of the sensorimotor cortex using functional magnetic resonance imaging (fMRI).

**Methods:**

In this prospective single-arm interventional study, 18 right-handed TBPI patients with chronic neuropathic pain were enrolled. All patients received a standardized MVF therapy (20 min/d) for 6 weeks. Pain intensity and psychological status were assessed using the Visual Analogue Scale (VAS), Self-Rating Anxiety Scale (SAS), Self-Rating Depression Scale (SDS), and Symptom Checklist 90 (SCL-90). Structural MRI and task-based fMRI with unilateral and bilateral hand motor tasks were performed at baseline and post-intervention to evaluate gray matter volume (GMV) and sensorimotor network activation. Twenty-five demographically matched healthy volunteers were recruited specifically as neuroimaging reference controls, having no role in the interventional comparison.

**Results:**

Visual Analogue Scale scores decreased significantly (5.917 ± 1.574 to 3.306 ± 2.860, *p* < 0.001) following MVF therapy, while psychological scales showed no marked changes. Post-treatment GMV increased in the superior temporal gyrus, parahippocampal gyrus, inferior frontal gyrus, and supplementary motor area (SMA). The movement of the affected hand resulted in increased activation in the contralateral primary motor and somatosensory cortices, showing a tendency toward a healthy activation pattern, particularly in non-dominant limb injury patients.

**Conclusion:**

Mirror visual feedback relieves TBPI-related chronic neuropathic pain and may partially normalize disordered sensorimotor cortical activation. Its analgesic effect is correlated with the recovery of sensorimotor cortical function, while no significant psychological improvements were seen over the short follow-up. As an easy, affordable non-invasive intervention, MVF exerts preliminary pain-relieving effects for refractory TBPI neuropathic pain.

## 1 Introduction

Traumatic total brachial plexus injuries (TBPI) are severe and devastating injuries involving upper extremity peripheral nerves, often resulting in severe and permanent upper limb dysfunction, particularly affecting young adults (1). Neuropathic pain is one of the most common and severe complications, occurring as moderate to severe pain in 30–80% of patients (2, 3). The condition may present with persistent burning or cramping pain in the affected limb, frequently accompanied by hyperalgesia and allodynia (4). This refractory pain responds poorly to conventional analgesics, severely impairing patients’ quality of life, hindering rehabilitation, and predisposing individuals to anxiety, depression, and other psychological disorders, which exacerbates the overall disease burden (5). The pathogenesis of pain in TBPI involves peripheral injury factors; however, accumulating evidence indicates that cortical functional reorganization and central sensitization constitute the core mechanisms underlying pain persistence and exacerbation (6–8). Compared with common peripheral nerve injuries, TBPI-induced central nervous system (CNS) plasticity exhibits distinct injury specificity. The significant structural and functional remodeling linked to root avulsion is a primary contributor to chronic intractable pain and constitutes a crucial target for clinical intervention in TBPI-related chronic pain (9).

Mirror visual feedback (MVF) therapy is a noninvasive therapeutic modality based on the principles of neuroplasticity. Its primary mechanism involves potentially correcting impaired cerebral cortical function, possibly attenuating aberrant sensorimotor cortical reorganization that occurs after nerve damage, and may interfere with central transmission of abnormal pain signals via visual illusions, thereby potentially alleviating denervation neuropathic pain (10, 11). In practice, the affected limb is positioned behind an opaque mirror, and the patient actively moves the unaffected limb while observing its mirrored reflection, creating the illusion of normal movement in the denervated limb to modulate cortical function. Neurophysiologically, this visual illusion may help activate the primary motor cortex (M1) corresponding to the denervated limb, delivers positive afferent feedback to the motor cortex, and could disrupt abnormal nociceptive input (12, 13), while synchronously engaging the sensorimotor cortical network (14). However, the precise and complete central neural regulatory pathway is yet to be established. Multiple clinical studies have validated that MVF may mitigate pathological sensorimotor cortical reorganization and produces sustained analgesia in patients with phantom limb pain (15), providing a theoretical foundation for its application in TBPI-associated pain.

The MVF therapy has been established to rely on neuroplasticity to alleviate neuropathic pain. Its foundational application targets post-amputation phantom limb pain, post-stroke spasticity and pain, complex regional pain syndrome (CRPS), and other conditions (16–21). Although preliminary investigations suggest that MVF relieves deafferentation pain after brachial plexus root avulsion, targeted intervention protocols and neuroimaging studies exploring its mechanisms in chronic neuropathic pain from traumatic TBPI remain scarce. Currently, there is no established standardized optimized MVF treatment regimen or objective efficacy evaluation system, which limits its precise clinical application.

To address these clinical needs and research gaps, the present study adapted and optimized the conventional MVF therapy protocol. Using functional magnetic resonance imaging (fMRI) as an objective neuroimaging measure, we investigated sensorimotor cortical functional reorganization in TBPI patients before and after MVF treatment in a prospective single-arm interventional study design. We thoroughly examined the clinical effectiveness and core mechanisms of MVF for chronic neuropathic pain in TBPI using clinical and psychological evaluations, such as the Visual Analogue Scale (VAS) and self-rating anxiety and depression scales. This study aims to provide a noninvasive, safe, and effective novel strategy for clinical management to address current knowledge limitations in this field.

## 2 Materials and methods

### 2.1 Participants

This prospective single-arm interventional study was performed to evaluate the clinical efficacy of MVF therapy in patients with neuropathic pain after TBPI and to explore the underlying central mechanisms. The study protocol was approved by the Ethics Committee of Guangxi Medical University (approval number: 2021[KY-E-328]) and was carried out in accordance with the Declaration of Helsinki. Written informed consent was obtained from all participants before enrollment after a full explanation of the study procedures.

Patients were enrolled according to the following inclusion criteria: (1) aged between 16 and 65 years; (2) diagnosed with unilateral TBPI of any etiology, a history of brachial plexus nerve release and/or nerve transposition surgery and no functional recovery of the affected limb; (3) right-handed; (4) neuropathic pain lasting more than 6 months, diagnosed in line with the International Association for the Study of Pain (IASP) criteria (22), and with a Visual Analog Scale (VAS) pain score ≥4; (5) normal brain structure on imaging with no evidence of concomitant brain injury; (6) no use of any effective analgesic medications during the study period (conventional analgesics are not effective to alleviate pain in the enrolled patients). The exclusion criteria were as follows: (1) history of CNS diseases; (2) severe psychiatric disorders, cognitive impairment, or poor compliance that would interfere with the completion of experimental tasks; (3) contraindications to MRI or excessive head motion during MRI scanning; (4) previous exposure to MVF therapy; (5) other chronic pain conditions either before or after TBPI; (6) other upper extremity neuropathic pain-related diseases, such as diabetes mellitus, multiple sclerosis, or post-stroke sequelae; (7) amputation of the affected limb for any reason; (8) ongoing treatment or recent adjustment of pain-related medications, including analgesics, anticonvulsants, antidepressants, or opioids; (9) concurrent rehabilitation or other pain-related interventions during the study period; (10) previous dorsal root entry zone (DREZ) lesioning or other neurosurgical procedures for pain management; (11) voluntarily withdrawing from the study.

Twenty-three consecutive eligible inpatients with unilateral TBPI-related chronic neuropathic pain were recruited from the Department of Orthopaedic Hand Surgery, First Affiliated Hospital of Guangxi Medical University. All eligible candidates were invited to participate in MVF therapy. Five candidates who had consented to participate in the study were later excluded: two of the candidates failed to maintain uninterrupted training for more than 1 week, and the other three used analgesics within the first training week. Eighteen patients completed the final study cohort. TBPI was diagnosed by senior attending physicians based on clinical manifestations, physical examination, electrophysiological results, imaging findings consistent with nerve root avulsion, and intraoperative observations. Out of the 18 patients, 17 were male, and 1 was female, with a mean age of 29.3 years (range, 16–53 years). The right limb was affected in 8 patients, while the left limb was affected in 10 patients. The interval from injury to the first fMRI scan ranged from 1 to 252 months (mean, 42.2 months). The baseline demographic and pain characteristics of the patients are summarized in Supplementary Tables 1–3.

An age- and sex-matched healthy control group consisting of 25 right-handed volunteers with no history of neurological, psychiatric, or chronic pain disorders was enrolled for neuroimaging comparison. These volunteers did not receive MVF therapy or undergo follow-up assessments. The control group consisted of 23 males and 2 females, with a mean age of 29.04 years (range, 21–40 years). All volunteers provided written informed consent before enrollment.

### 2.2 Experimental task design

**Figure 1:**
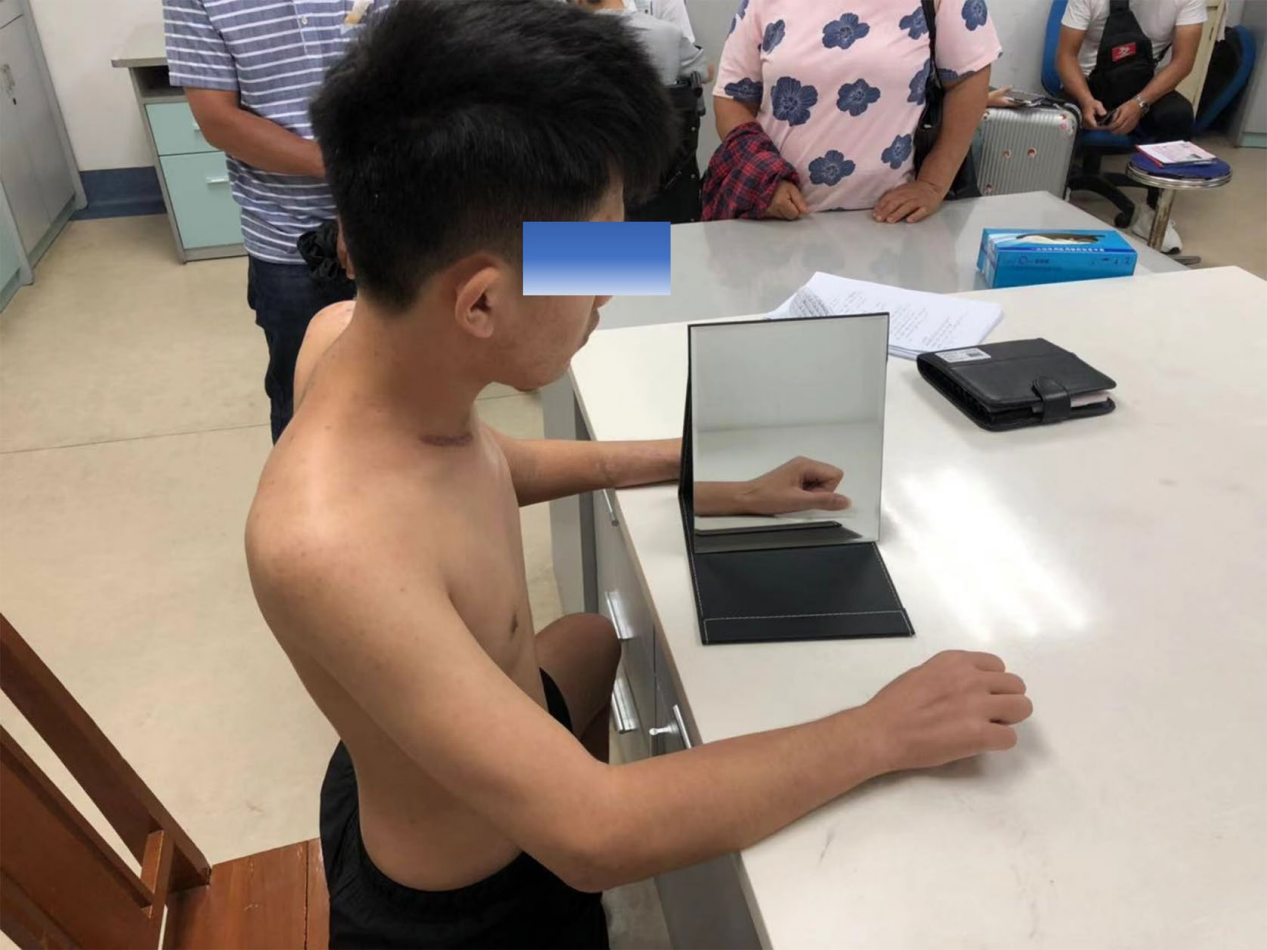
MVF training session.

Participants first completed basic demographic information registration and reported their pain scores, pain frequency, pain characteristics, and pain duration, followed by a standardized psychological questionnaire assessment. Before treatment, all participants underwent brain fMRI (BOLD sequence) scanning in the order of recruitment, including three-dimensional T1-weighted structural imaging and task-state fMRI data acquisition during left-hand motor, right-hand motor, and bimanual motor tasks. Researchers explained and demonstrated the procedures of MVF therapy to participants; the MVF exercise was taught during the first outpatient visit, utilizing physical aids and direct demonstrations by clinicians. All participants maintained contact with the research team via a unified social media platform equipped with standardized tutorial videos to standardize home-based training. Prior to independent home training initiation, every participant submitted a video recording of their first self-training session. The research team reviewed each submitted video, delivered personalized corrective feedback, and validated standardized training operation before participants proceeded with unsupervised home practice. Daily follow-up communications were arranged within the initial 2 days of home training to correct improper movements and clarify any patient inquiries. Subsequent routine follow-ups were scheduled biweekly, with additional real-time communication available whenever participants reached out for troubleshooting or guidance. Regular check-in messages were sent throughout the entire intervention period to track training adherence, collect self-reported training completion frequency, record training experience, and gather pain-related subjective feedback. While formal written training logs were not mandatory, participants were encouraged to reach out to the research team proactively at any time to report training obstacles, procedural inquiries, or fluctuating symptoms. The detailed protocol of MVF therapy was as follows: in a quiet environment, participants used a portable folding mirror and placed the affected hand behind the mirror to be concealed; they then watched the reflection of the unaffected hand in the mirror and performed motor training including hand opening and closing, grasping, finger-to-finger opposition, wrist flexion and extension, as well as sensory training such as touching a towel or a sensory training ball. Both hands were required to perform the same motor simultaneously, with the affected limb performing imagined movements; participants were instructed not to reveal their faces in front of the mirror during scanning and treatment (Figure 1). The intervention lasted for 6 weeks, with one training session per day and 20 min per session. After the intervention, fMRI examination was repeated, pain scores were re-evaluated, and pain frequency, characteristics, and duration were recorded using the same psychological questionnaires.

The task-state fMRI experiment was designed as a block design using E-Prime software. Each participant completed three tasks: left-hand motor, right-hand motor, and bimanual motor. Each task consisted of 3 repeated blocks, and each block included a 30-s task period and a 30-s rest period. Before each task, a fixation cross “+” was positioned on the screen for 12 s for baseline preparation and signal stabilization. Then, an animation of hand grasping and opening was displayed, and participants were instructed to perform the same motor actions synchronously at the frequency of the animation for 30 s, followed by a 30-s rest period. Each motor task was repeated 3 times. The default task sequence was left-hand motor → right-hand motor → bimanual motor. For participants with right-limb injury, the sequence was adjusted to right-hand motor → left-hand motor → bimanual motor to avoid interference from unaffected hand motors on the activation of the affected hemisphere. If participants were unable to move the affected hand due to motor dysfunction, they were instructed to observe the motor frequency on the screen and perform motor imagery of the affected hand only. All task procedures were explained in detail to participants before they entered the MRI scanning room, and practice sessions were conducted on the scanning table prior to formal scanning to ensure full understanding and accurate performance. During scanning, examiners visually monitored participants’ task performance in real time, including actual movements and/or mirror movements. If participants failed to perform the task correctly during scanning, immediate correction was provided, and scanning was repeated. After scanning, participants were asked to reconfirm the motor tasks they had completed to ensure the accuracy and reliability of task performance.

### 2.3 MRI data acquisition

**Figure 2:**
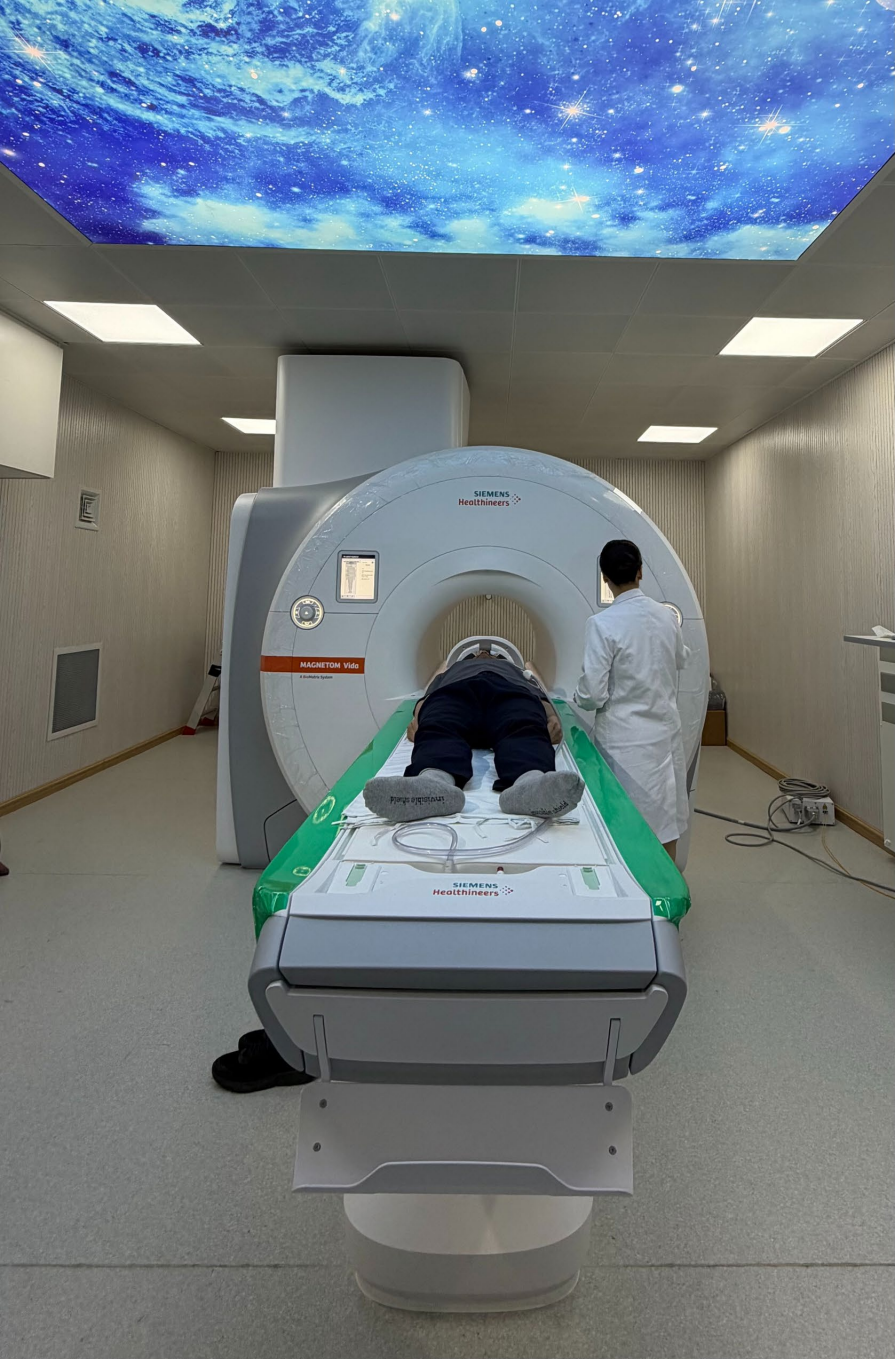
The 3.0 T MRI scanner (PRISMA, Siemens) used in this study.

Data acquisition was performed using a PRISMA 3.0T MRI scanner (Figure 2) equipped with a 64-channel head coil (Siemens). Participants were positioned supine on the MRI bed, with inflatable pillows placed on both sides of the head to minimize head motion during scanning. Participants were advised to stay motionless throughout the image acquisition process, except during necessary hand movements needed to complete the performance.

### 2.4 Three-dimensional (3D) whole-brain structural imaging scanning

Axial 3D whole-brain structural images were acquired using a T1-weighted sequence with the 3D magnetization-prepared rapid gradient-echo (3D MP-RAGE) technique. The scanning parameters were set as follows: repetition time (TR) = 2,300 ms, echo time (TE) = 2.98 ms, flip angle = 9°, field of view (FOV) = 256 × 256 mm, slice thickness = 1 mm, voxel size = 1.0 × 1.0 × 1.0 mm, and total number of slices = 176.

### 2.5 Task-related fMRI scanning

Task-mode functional images were scanned using the following parameters: TR = 2000 ms, TE = 35.0 ms, flip angle = 90°, number of averages = 1, FOV = 240 × 240 mm, slice thickness = 3 mm, voxel size = 3.0 × 3.0 × 3.0 mm, and total scanning duration = 192 s.

### 2.6 fMRI data processing

Functional images were preprocessed using DPABI V4.2 following standard preprocessing pipelines. The preprocessing procedures included the removal of the first 10 volumes to eliminate unstable initial scanning signals, slice timing correction (40 slices acquired in interleaved ascending-descending order: 2, 4, 6, …, 40, 1, 3, 5, …, 39, with the 40th slice set as the reference slice), head motion realignment, co-registration of echo-planar imaging (EPI) images to individual T1-weighted structural images, spatial normalization to the Montreal Neurological Institute (MNI) standard space using the T1 New Segment + DARTEL algorithm with a resampled voxel size of 3 × 3 × 3 mm, and spatial smoothing using a Gaussian kernel with a full width at half maximum (FWHM) of 8 mm.

### 2.7 Parameter estimation and statistical analysis of imaging data

#### 2.7.1 First-level general linear model (GLM) analysis

For each participant, a voxel-wise first-level GLM was constructed using SPM12. Each scanning run consisted of three sessions corresponding to the left stimulation (L), right stimulation (R), and bilateral stimulation (BOTH) conditions, with 90 volumes acquired per session. A block-related design was adopted, with stimulus onsets occurring at 1st, 31st, and 61st scans and a block duration of 15 scans. Six head motion parameters derived from realignment correction were incorporated as nuisance regressors to control for head motion artifacts. A high-pass filter with a cutoff frequency of 128 s and a first-order autoregressive [AR(1)] model for temporal autocorrelation correction were applied to preprocess time-series data. Four contrast maps were generated for subsequent analyses, including L, R, and BOTH conditions, and the average activation across the three experimental conditions.

#### 2.7.2 Second-level analysis

Individual contrast images from first-level GLM analysis were imported into a second-level flexible factorial model via the GLM_Flex_Fast4 toolbox. The factorial model included Group, Condition1, Condition2, and Repeat as fixed factors, with subjects defined as random effects nested within the interaction of Condition1 × Condition2 × Repeat. Age, gender, and time since injury were included as nuisance covariates to eliminate confounding effects. The standard DPABI brain mask (BrainMask_05_61 × 73 × 61) was applied to restrict analyses to brain tissues, and spatial smoothing parameters were empirically estimated during model estimation.

#### 2.7.3 Statistical inference

Whole-brain voxel-wise statistical analysis was performed with an uncorrected voxel-level height threshold of *p* < 0.001. No cluster extent threshold was imposed in the initial thresholding procedure (*k* = 0 voxels). To control type I error inflation induced by multiple comparisons, statistically significant brain clusters were identified and reported only after surviving cluster-level false discovery rate (FDR) correction at *p* < 0.001. Additionally, both cluster-level and peak-level family-wise error (FWE) and FDR-corrected statistical values were calculated.

#### 2.7.4 Region of interest (ROI) analysis and quality control

The ROI segmentation was performed based on the Automated Anatomical Labeling (AAL) 90 brain atlas. Mean parameter estimates and contrast values within each predefined ROI were extracted for subsequent visualization and auxiliary statistical analyses. Strict quality control was implemented for all imaging data. Visual inspection and quantitative verification were conducted for head motion realignment parameters, framewise displacement, and MNI spatial normalization quality. All patients met the predefined head motion criteria with translational and rotational motion less than 3 mm and 3°, respectively. Nuisance motion regressors were included in all first-level GLMs to further eliminate residual motion-related artifacts.

### 2.8 Clinical evaluation

All patients were evaluated using the Visual Analogue Scale (VAS), Self-Rating Anxiety Scale (SAS), Self-Rating Depression Scale (SDS), and Symptom Checklist 90 (SCL-90). Patients with a VAS score ≥4 were included in the relevant analyses. Sleep-related outcomes were assessed through structured follow-up interviews instead of standardized sleep questionnaires. The survey addressed participants’ difficulties in falling asleep, the incidence of pain-induced awakenings during the night, the approximate frequency of these awakenings, and their subjective evaluations of sleep quality changes after treatment. Patients were also asked to assess the post-treatment changes in pain-related sleep disturbances as improved, stable, or deteriorated. Additionally, patients’ pre- and post-treatment subjective pain perceptions were obtained through interviews and documented as qualitative narratives to supplement quantitative outcome indicators.

### 2.9 Statistical analysis

Clinical and psychological data were analyzed using SPSS software. Continuous variables are expressed as mean ± standard deviation (SD). Paired *t*-tests were used for comparisons before and after treatment; nonparametric tests were applied when the data did not meet the assumptions of normality. Spearman correlation analyses were used to explore the correlations between changes in pain intensity (assessed by VAS) and neuroimaging measures. A two-tailed *p*-value <0.05 was considered statistically significant.

Given the limited sample size and unbalanced group sizes of the intervention group, Hedges’ g (a bias-corrected variant of Cohen’s d for small samples) was used to calculate effect sizes for ROI activation value comparisons. The applied correction formula was $g=d\cdot (1-\frac{3}{4\mathrm{df}-1})$ , where *df* = 29. 95% confidence intervals (CIs) for Hedges’ *g* were estimated using the non-central *t*-distribution approach, which provides improved accuracy under small-sample conditions.

## 3 Results

All 18 patients underwent 6-week MVF therapy and completed pre- and post-treatment VAS, SAS, SDS, and SCL-90 assessments. Two patients (Nos. 10, 16) refused to receive follow-up fMRI scans. Another three patients (Nos. 3, 6, 8) were excluded from statistical analysis owing to poor-quality task-state fMRI images, and abnormal T1-weighted structural images were identified in two of these three patients. Consequently, final statistical analyses were performed on T1 structural data from 14 patients and task-state fMRI data from 13 patients.

### 3.1 Changes in subjective pain perception following MVF treatment

After 6 weeks of MVF therapy, out of the 18 patients, five (Nos. 1, 3, 6, 15, 16) reported no obvious pain relief or only a slight increase in pain, three (Nos. 8, 10, 11) reported mild pain improvement, while the other 10 patients reported significant pain reduction. Detailed changes in pain perception are summarized in Table 1. In terms of sleep status, 3 patients (Nos. 4, 9, 12) reported improved sleep quality after intervention, while 3 patients (Nos. 8, 15, 17) experienced sleep disturbances, and the other 12 patients showed no obvious changes in sleep.

**Table 1:** Subjective changes in pain perception in TBPI patients following MVF treatment.

Patient no.	VAS scores	Changes in pain perception	Sleep impact
1	5	Pain was slightly reduced, no change in the nature of pain, but occurring less frequent, approximately 1–2 times a month; the illusion of the ipsilateral upper limb being touched when the affected cheek was touched disappeared	Normal
2	2	The affected limb felt lighter, less frequent pain, approximately 5–6 times per month	Normal
3	9	No significant change in pain, either in pain intensity or frequency	Normal
4	0	Complete pain relief	Significant improvement in sleep quality
5	3	Significant reduction in pain intensity, with the frequency of pain decreasing from every 10 min to only one or two times per day	Normal
6	5.5	No significant change in pain, either in pain intensity or frequency	Normal
7	0	Complete pain relief	Normal
8	5.5	Pain was reduced, and nocturnal pain did not occur. Touching the affected side of the patient’s face still had the phantom sensation of the affected wrist and forearm	Affect sleep
9	0	The pain was felt more intensely during the MVF therapy, but after 6 weeks of therapy, the pain intensity decreased to an ant bite-like pain. The frequency of pain was reduced by 30–40%. Self-perception of less sweating in the affected limb than before	Improved sleep quality
10	5.5	The pain is still present, but it occurs less frequently and lasts shorter. Pain occurs multiple times a day for 10 s or so each time	Normal
11	5.5	Little change in the frequency of pain intensity, but relief of pain in the affected hand	Normal
12	2	The pain score was reduced to a minimum of 2, and the number of nighttime awakenings was reduced to about 7–8 per day, but the pain score was still 7 at the time of awakening	Improved sleep quality
13	2	Pain has been reduced	Normal
14	2	Feeling more strength in the muscles of the affected upper limb and slight relief of pain	Normal
15	7	The intensity of pain remained unchanged but occurred significantly less frequently (once a week, lasting 1–2 min each time), and the patient experienced phantom motor sensations in the affected hand during training	Affect sleep
16	7	The pain is of the same nature as before and lasts for a few seconds at a time. The range of pain decreased from the entire upper extremity to pain only distal to the forearm. The pain was not present during the mirror image training and recurred after stopping the training, while the upper limb was felt to be fuller and stronger, and the illusion of touching the affected cheek on the outer side of the affected upper arm was experienced	Normal
17	0	Generally, there is no pain, with only occasional mild pain occuring1-2 times a month	Affect sleep
18	0	The pain is obvious when practicing, but not so much in daily life. The patient feels that they can move the affected hand a little more easily after practicing	Normal

TBPI, total brachial plexus injury; MVF, mirror visual feedback; VAS, Visual Analogue Scale.

### 3.2 Changes in gray matter volume following MVF treatment

**Figure 3:**
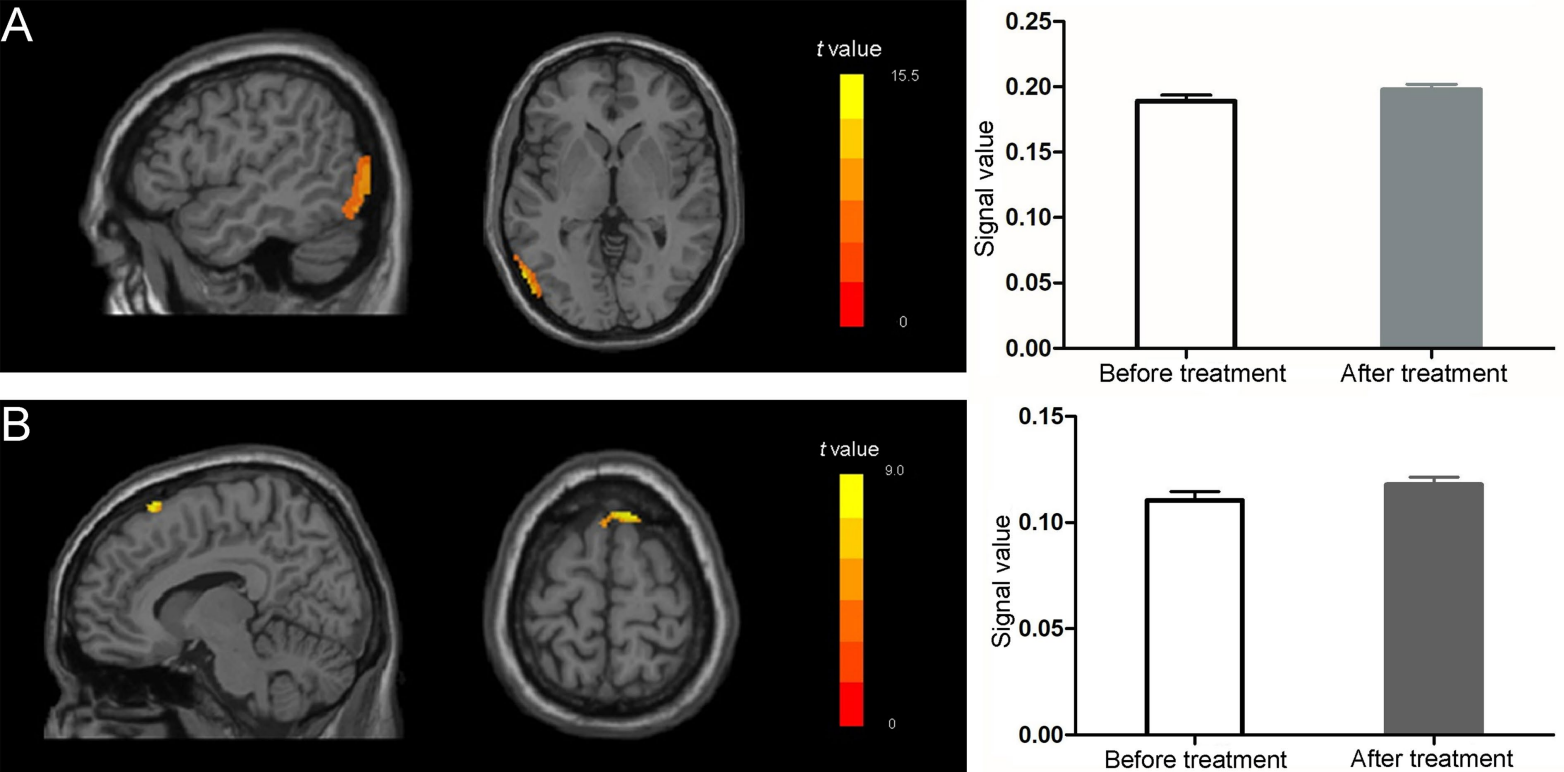
Brain maps of gray matter volume changes following MVF therapy. **(A)** Superior temporal gyrus/parahippocampal gyrus/inferior frontal gyrus (MNI coordinates: −58.5, −69, 7.5). **(B)** Supplementary motor cortex (MNI coordinates: 9, 25.5, 67.5). The left panels show the sagittal and coronal views of brain regions with significant gray matter volume differences, respectively. The right panels display the mean contrast values of the corresponding differential brain regions across groups and conditions. Red indicates positive differences.

We selected brain regions showing significant differences in gray matter volume between control and patient groups in our prior study (23) as ROIs; these included the bilateral parahippocampa gyrus, superior temporal gyrus, paracentral lobule, postcentral gyrus, precentral gyrus, middle frontal gyrus, inferior frontal gyrus, supplementary motor area, middle occipital gyrus, superior parietal lobule, cuneus, right middle temporal gyrus, right inferior temporal gyrus, left precuneus, insula, pons, cingulate gyrus, inferior parietal lobule, thalamus, and globus pallidus, to assess changes in gray matter volume before and after treatment. The changes in gray matter volume within these ROIs may be associated with chronic neuropathic pain in patients following total brachial plexus avulsion. All statistical comparisons across these ROIs were corrected using cluster-level FDR correction (*p* < 0.001), and the correction strategy was applied consistently throughout all analyses. Relative to baseline, we observed a significant increase in gray matter volume in the superior temporal gyrus/parahippocampal gyrus/inferior frontal gyrus, and supplementary motor cortex; no significant differences were detected in other brain regions (Figure 3).

### 3.3 Changes of brain activation during hand motor tasks following MVF therapy

Task-state fMRI analyses were performed on data from 16 patients. Two patients (Nos. 10 and 16) were excluded due to incomplete post-treatment data. For ROI analyses, brain regions functionally activated during hand motor tasks, including the bilateral precentral gyri, bilateral postcentral gyri, and supplementary motor areas (SMAs), were predefined. Activation magnitudes within these ROIs were extracted using DPABI software. Paired sample *t*-tests were adopted to compare brain activation before and after treatment, and independent-samples *t*-tests were used to examine intergroup differences between patients and healthy controls.

For cluster-level analyses, three additional patients (Nos. 3, 6, and 8) were excluded due to suboptimal image quality. The remaining 13 patients were stratified into two groups based on the side of brachial plexus injury: left TBPI group (*n* = 6) and right brachial plexus injury (BPI) group (*n* = 7). Intergroup comparisons between patient subgroups and healthy controls were conducted to evaluate alterations in brain activation during unimanual left-hand, unimanual right-hand, and bimanual motor tasks pre- and post-treatment.

Following MVF therapy, patients in the left TBPI group exhibited significantly increased activation in the right precentral and postcentral gyri during the affected-side (left-hand) motor task relative to baseline (Table 2). No significant pre-to-post treatment differences in ROI activation were observed during right-hand or bimanual motor tasks.

**Table 2:** ROI activation during left-hand motor task in the left TBPI group between pre- and post-treatment (*n* = 6).

Pair (post – pre)	Mean ± SD	*t*	Hedges’ *g* (95% CI)	*P*
Left precentral gyrus	0.376 ± 1.142	0.988	0.340 [−0.380, 1.029]	0.352
Right precentral gyrus	0.639 ± 0.746	2.572	0.884 [0.0003, 1.718]	0.033
Left SMA	0.631 ± 1.120	1.689	0.581 [−0.200, 1.318]	0.130
Right SMA	0.572 ± 0.864	1.986	0.683 [−0.130, 1.449]	0.082
Left postcentral gyrus	0.097 ± 1.381	0.210	0.072 [−0.607, 0.744]	0.839
Right postcentral gyrus	0.579 ± 0.698	2.488	0.855 [−0.018, 1.679]	0.038

Paired *t*-tests were used to compare pre- and post-treatment values.

ROI, region of interest; TBPI, total brachial plexus injury; SD, standard deviation; SMA, supplementary motor area.

Independent-samples *t*-tests were used to compare ROI activation between the left TBPI group and healthy controls at baseline and post-treatment, respectively. During the left-hand motor task, patients showed significantly lower activation in the right precentral and postcentral gyri at baseline relative to healthy controls; however, these between-group differences became insignificant after MVF therapy. By contrast, left and right SMA activation showed no between-group differences at baseline, but was significantly greater in patients after treatment than in healthy controls (Table 3). During the right-hand motor task, patients exhibited significantly greater activation in the right postcentral gyrus and right SMA at baseline relative to healthy controls, with no significant between-group differences observed post-treatment (Table 4). No other significant between-group differences in ROI activation were detected before or after treatment in the remaining brain regions. During bimanual motor tasks, no significant differences in ROI activation were found between the left TBPI group and healthy controls at either time point.

**Table 3:** Activation values of left-hand motor tasks in the left TBPI group and healthy controls before and after treatment (*n* = 6).

Group	*F*	Significance	*t*	Hedges’ *g* (95% CI)	*P*
Right precentral gyrus (pre-treatment)	2.825	0.103	−2.125	−0.941 [−1.834, −0.033]	0.041
Right precentral gyrus (post-treatment)	0.445	0.509	−0.381	−0.169 [−1.036, 0.702]	0.706
Right postcentral gyrus (pre-treatment)	0.203	0.655	−2.220	−0.983 [−1.879, −0.072]	0.034
Right postcentral gyrus (post-treatment)	0.004	0.949	−0.115	−0.051 [−0.918, 0.817]	0.909
Left SMA (pre-treatment)	3.068	0.089	−0.358	−0.159 [−1.026, 0.712]	0.723
Left SMA (post-treatment)	1.761	0.194	2.987	1.322 [0.381, 2.244]	0.005
Right SMA (pre-treatment)	1.733	0.197	0.153	0.068 [−0.801, 0.935]	0.880
Right SMA (post-treatment)	0.310	0.582	3.863	1.710 [0.726, 2.671]	<0.001

*F* and Significance values represent the results of Levene’s test for equality of variances. All Significance values >0.05, confirming homogeneity of variances.

TBPI, total brachial plexus injury; SMA, supplementary motor area.

**Table 4:** Activation values of right-hand motor tasks in the left TBPI group and healthy controls before and after treatment (*n* = 6).

Group	*F*	Significance	*t*	Hedges’ *g* (95% CI)	*P*
Right postcentral gyrus (pre-treatment)	1.952	0.172	2.147	0.951 [0.042, 1.844]	0.039
Right SMA (pre-treatment)	3.705	0.063	2.348	1.040 [0.124, 1.939]	0.023

*F* and Significance values represent the results of Levene’s test for equality of variances. All Significance values >0.05, confirming homogeneity of variances.

TBPI, total brachial plexus injury; SMA, supplementary motor area.

Patients in the right TBPI group exhibited a significant reduction in activation of the right SMA during the left-hand motor task (healthy side) following MVF therapy, relative to baseline (Table 5). No significant pre-to-post treatment differences in activation were observed for the remaining motor tasks.

**Table 5:** ROI activation during left-hand motor task in the right TBPI group between pre- and post-treatment (*n* = 7).

Pair (post – pre)	Mean ± SD	*t*	Hedges’ *g* (95% CI)	*P*
Left precentral gyrus	−0.137 ± 0.786	−0.462	−0.152 [−0.898, 0.585]	0.661
Right precentral gyrus	0.133 ± 1.182	0.297	0.098 [−0.640, 0.834]	0.777
Left SMA	−0.302 ± 0.496	−1.610	−0.529 [−1.318, 0.241]	0.159
Right SMA	−0.413 ± 0.253	−4.313	−1.418 [−2.481, −0.289]	0.005
Left postcentral gyrus	0.074 ± 0.571	0.342	0.112 [−0.626, 0.849]	0.744
Right postcentral gyrus	−0.181 ± 1.208	−0.397	−0.131 [−0.874, 0.607]	0.705

Paired *t*-tests were used to compare pre- and post-treatment values.

ROI, region of interest; TBPI, total brachial plexus injury; SD, standard deviation; SMA, supplementary motor area.

Independent-samples *t*-tests were performed to compare ROI activation values between the right TBPI group and healthy controls before and after treatment, respectively. During the affected-side (right-hand) motor task, the right TBPI group exhibited significantly lower activation in the left precentral and postcentral gyri at baseline relative to healthy controls, whereas these between-group differences disappeared after treatment. By contrast, activation in the right postcentral gyrus was significantly lower in the right TBPI group than in healthy controls only after treatment, with no significant difference observed at baseline (Table 6). During left-hand and bimanual motor tasks, no significant between-group differences in ROI activation were detected between the right TBPI group and healthy controls either before or after treatment.

**Table 6:** Activation values of left-hand motor tasks in the right TBPI group and healthy controls before and after treatment (*n* = 7).

Group	*F*	Significance	*t*	Hedges’ *g* (95% CI)	*P*
Left precentral gyrus (pre-treatment)	0.262	0.613	−2.484	−1.044 [−1.917, −0.150]	0.019
Left postcentral gyrus (pre-treatment)	0.002	0.968	−2.413	−1.014 [−1.886, −0.123]	0.022
Right postcentral gyrus (post-treatment)	0.006	0.941	−2.079	−0.873 [−1.730, −0.001]	0.046

*F* and Significance values represent the results of Levene’s test for equality of variances. All Significance values >0.05, confirming homogeneity of variances.

TBPI, total brachial plexus injury.

### 3.4 Cluster-level analysis

After data normalization using SPM, group-level brain activation maps for hand motor tasks were generated (Supplementary Figures 1–3). Mixed repeated-measures ANOVA revealed no significant group × time × task interaction, and no significant differential activation clusters between groups before and after treatment.

### 3.5 Changes in VAS score

Paired-samples *t*-tests conducted on 18 patients demonstrated a significant difference in VAS scores before versus after treatment (*p* < 0.001). Specifically, VAS pain scores were significantly decreased following MVF therapy (Table 7).

**Table 7:** Comparison of VAS, SAS, SDS, and SCL-90 scores before and after treatment (*n* = 18).

Variables	Pre-treatment (Mean ± SD)	Post-treatment (Mean ± SD)	Hedges’ *g* (95% CI)	*t*	*P*
VAS	5.917 ± 1.574	3.306 ± 2.860	1.065 [0.477, 1.653]	4.730	<0.001
SAS	39.653 ± 8.551	37.819 ± 7.058	0.277 [−0.195, 0.749]	1.231	0.235
SDS	42.708 ± 8.092	42.375 ± 8.468	0.042 [−0.420, 0.505]	0.189	0.853
SCL-90	1.523 ± 0.456	1.461 ± 0.455	0.337 [−0.139, 0.814]	1.499	0.152

Paired *t*-tests were used to compare pre- and post-treatment values.

VAS, Visual Analogue Scale; SAS, Self-Rating Anxiety Scale; SDS, Self-Rating Depression Scale; SCL-90, Symptom Checklist 90; SD, standard deviation.

### 3.6 Psychological questionnaire

Psychological questionnaire assessments were performed in 18 patients before and after MVF therapy, including the Self-Rating Anxiety Scale (SAS), Self-Rating Depression Scale (SDS), and Symptom Checklist 90 (SCL-90). Before treatment, SAS scores ranged from 28.75 to 65.00 (mean = 39.653), and after treatment, SAS scores ranged from 28.75 to 56.25 (mean = 37.819). SDS scores were 31.25–63.75 (mean = 42.708) at baseline and 32.50–60.00 (mean = 42.375) post-treatment. The total symptom index of SCL-90 was 1.03–2.63 (mean = 1.523) before treatment and 1.03–2.48 (mean = 1.461) after treatment. Paired-samples *t*-tests indicated no statistical changes in these psychological scale scores between baseline and post-treatment assessments within the current sample and follow-up duration (Table 7). Spearman’s correlation analysis revealed that baseline and post-treatment psychological scores (SAS, SDS, and SCL-90 total symptom index) were positively correlated with each other at both time points. For pain-related correlations, while baseline VAS scores were not associated with any psychological measures, post-treatment VAS scores showed a positive correlation with post-treatment SAS scores (*r* = 0.451, *p* = 0.006). A correlation was also observed between pre- and post-treatment VAS scores (*r* = 0.623, *p* = 0.006) (Table 8). These correlational findings should be interpreted with caution due to the limited sample size.

**Table 8:** Spearman’s correlation analysis results between psychological scale scores and VAS scores (*n* = 18).

Variable	SAS pre	SAS post	SDS pre	SDS post	SCL-90 pre	SCL-90 post	VAS pre	VAS post
SAS pre	1 (−)							
SAS post	0.441 (0.067)	1 (−)						
SDS pre	0.520 (0.027*)	0.306 (0.218)	0.707 (0.001**)					
SDS post	0.009 (0.971)	0.618 (0.006*)	0.306 (0.217)	1 (−)				
SCL-90 pre	0.490 (0.039*)	0.134 (0.595)	1 (−)	0.306 (0.217)	1 (−)			
SCL-90 post	0.260 (0.297)	0.472 (0.048*)	0.604 (0.008**)	0.592 (0.010**)	0.604 (0.008**)	1 (−)		
VAS pre	0.090 (0.724)	0.321 (0.195)	−0.107 (0.672)	0.122 (0.630)	−0.107 (0.672)	0.199 (0.428)	1 (−)	
VAS post	−0.131 (0.604)	0.451 (0.006)	−0.190 (0.451)	0.403 (0.098)	−0.190 (0.451)	0.123 (0.626)	0.623 (0.006**)	1 (−)

Values are Spearman’s correlation coefficients (*r*) with *p*-values in parentheses. *p < 0.05, **p < 0.01.

VAS, Visual Analogue Scale; SAS, Self-Rating Anxiety Scale; SDS, Self-Rating Depression Scale; SCL-90, Symptom Checklist 90.

## 4 Discussion

### 4.1 Cortical structural and functional abnormalities in TBPI patients with neuropathic pain

Studies in patients with neuropathic pain following traumatic TBPI have demonstrated extensive reductions in gray matter volume within the sensorimotor cortex. These structural alterations may be closely associated with the onset and severity of pain, a finding consistent with the conclusions of two meta-analyses on chronic neuropathic pain (24, 25), further suggesting shared pathological features of the CNS in chronic pain states. Most TBPI patients present with varying degrees of nerve root avulsion, predominantly involving the lower trunk. Such injury directly triggers extensive denervation-induced degeneration in the sensorimotor cortex of the brain. Given the large cortical representation of hand sensorimotor function, damage often results in a functional deficit region; even after surgical nerve repair, complete restoration of normal function remains challenging (26). Consistent with the task-based fMRI results in our study, patients with post-traumatic pain exhibited significantly decreased activation in the precentral and postcentral gyri during affected hand movement pretreatment compared with healthy controls. The primary mechanism underlying this observation likely involves the loss of sensorimotor function in the affected limb, particularly the hand, suggesting impaired afferent sensorimotor signaling in the corresponding cerebral cortex. Notably, these deafferented regions, especially the sensory cortex, represent key nodes in pain modulation. Their functional impairment may create conditions for invasion and maladaptive reorganization of adjacent normally functioning cortical areas, a pattern observed in Patients 1, 8, 14, 15, and 18 in our study. Collectively, differentiation of sensorimotor signaling and maladaptive cortical reorganization may jointly contribute to the initiation and maintenance of neuropathic pain following traumatic TBPI.

These structural and functional changes in the cerebral cortex essentially reflect CNS neuroplasticity, defined as the ability of the CNS to dynamically adjust its structural organization and functional state in response to functional demands (27). Neuroplasticity is not exclusive to neural injury; the brain also undergoes dynamic remodeling under physiological conditions. Physiological adaptive processes such as behavior-related stimulation and motor learning can induce an expanded representational area of the trained regions in the motor cortex (28, 29).

### 4.2 Mechanism and therapeutic effect of MVF therapy for TBPI neuropathic pain

The MVF therapy is based on the mechanism where a mirror reflection is used to reflect movements of the unaffected limb onto the affected side, creating a visual illusion of intact function in the affected limb, thereby potentially alleviating pain and improving limb function. At present, this therapy has been widely applied in the clinical treatment of phantom limb pain after amputation and motor dysfunction after stroke (16–19).

Accumulating evidence has suggested that the analgesic effect of MVF therapy is directly associated with enhanced activation of the primary motor cortex (M1) in the affected upper limb (12, 13). In task-based fMRI studies, voluntary manual movement can specifically activate the contralateral sensorimotor cortex. This task exhibits strong activation signals, excellent reproducibility, and minimal confounding factors, representing an ideal task design for our study. Our results demonstrated that after 6 weeks of MVF therapy, the mean fMRI activation values in the precentral and postcentral gyri corresponding to the affected side were not significantly different from those in healthy controls (whereas values were significantly lower than those in controls before treatment), indirectly implying that MVF therapy may help activate the affected M1. The improved activation of the primary somatosensory cortex (S1) may suggest enhanced interactions between adjacent brain functional regions. Substantial evidence indicates that stimulation of the M1 and prefrontal cortex activates brain regions involved in pain integration and modulation. Epidural electrical stimulation of M1 increases cerebral blood flow in pain-related regions, including the lateral/medial thalamus and anterior cingulate cortex (ACC) (30). Motor cortex stimulation exerts an inhibitory effect on nociceptive neurons in the thalamus and spinal cord (31). Stimulation of M1 also reduces opioid receptor availability in the ACC and periaqueductal gray (PAG), a change significantly correlated with pain relief (32). Furthermore, MVF-induced increases in PAG activity are associated with decreased local gamma-aminobutyric acid (GABA) activity (33). The PAG modulates nociceptive input via the rostral ventromedial medulla (RVM), which further projects to the dorsal horn of the spinal cord, forming the descending nociceptive modulatory system and potentially exerting analgesic effects (34).

Our study found that in patients with left TBPI, MVF therapy significantly increased activation in the precentral and postcentral gyri of the affected hemisphere compared with pretreatment levels. Moreover, activation of the bilateral SMA was significantly higher than in healthy controls, which may be related to the activation of mirror neurons. Mirror neurons were first identified in area F5 of the ventral premotor cortex in macaques; they fire during both execution and observation of goal-directed hand movements and mediate the coupling of action execution and observation through relevant cortical circuits (35). The core network of the human mirror neuron system (MNS) mainly includes the inferior frontal gyrus (IFG), inferior parietal lobule (IPL), and superior temporal sulcus (STS); MVF therapy could activate this network, which may help improve the function of the affected limb (36). Rehabilitation strategies such as motor imagery also include neuroplasticity and improve upper limb function via goal-directed motor tasks (37). Studies have confirmed that the human M1 exhibits mirror neuron-like properties, with movement-specific and lateralized activation during action observation (38, 39). A magnetoencephalography study (40) reported that MVF therapy modulates M1 activity and alters connectivity within the superior temporal gyrus-related perceptual-attentional network, supporting the potential mediating role of the MNS.

### 4.3 Lateralized differences in MVF therapy effects

Compared with the left TBPI group (showing significant differences in activation of the ipsilesional precentral and postcentral gyri, as well as bilateral anterior cerebellar lobes before and after treatment), the right TBPI group exhibited no significant changes in activation of the ipsilesional precentral and postcentral gyri between pre- and post-treatment. Significant reductions in activation of the right anterior cerebellar lobe were observed only during motor tasks of the contralesional (left-handed) hand after treatment. Moreover, activation of the right postcentral gyrus during motor tasks of the ipsilesional (right-handed) hand was significantly lower in the right TBPI group than in healthy controls after treatment. Cluster analysis revealed no significant task-related differences between groups.

These findings may be partially interpreted in the context of cerebral functional lateralization. Previous fMRI studies have demonstrated stronger activation and larger cortical representation in the primary motor cortex (M1) contralateral to the dominant hand (41). Cortical reorganization induced by injury to the dominant upper limb is significantly more extensive than that caused by injury to the non-dominant limb (42). All patients in our study were right-handed. We hypothesize that, under identical MVF therapy, the cortex controlling the non-dominant (left) hand is more prone to exhibit significant activation. Alternatively, MVF therapy may reduce the demand for functional compensation in the hemisphere corresponding to the non-dominant hand, thereby possibly facilitating the re-establishment of interhemispheric inhibition, improving functional balance between brain regions, and potentially optimizing cortical reorganization in the dominant hemisphere. These hypotheses warrant further validation in future studies.

### 4.4 Clinical efficacy and comprehensive outcome of MVF therapy

Following MVF therapy, only 1 patient (No. 16) showed an increase in VAS score, while 4 patients (Nos. 3, 6, 11, and 15) exhibited no notable change in VAS score. No statistical analysis was conducted for individual cases. The majority of patients demonstrated various degrees of improvement in VAS score, subjective pain intensity, and pain frequency. Notably, among these 5 patients with unchanged or increased VAS scores, 3 patients (Nos. 11, 15, and 16) reported marked reductions in pain frequency and severity, as well as significant improvements in limb mobility and motor function. As pain is a complex, multifactorial subjective experience with substantial individual variability, self-report remains the gold standard for pain assessment. Even in patients with minimal changes in VAS score, improvements in individual subjective perception and significant reductions in group-level VAS scores after treatment collectively suggest that MVF therapy may alleviate intractable neuropathic pain in patients with TBPI. This therapeutic effect was also reflected in changes in cerebral gray matter volume: patients exhibited significantly increased gray matter volume in the superior temporal gyrus, parahippocampal gyrus, inferior frontal gyrus, and supplementary motor cortex following treatment. The human inferior frontal gyrus (Broca’s area) is involved in both phonological representation and hand motor representation. Our previous research (23) has shown that increased gray matter volume in this region may correlate with MVF-induced mirror neuron activation and amelioration of maladaptive reorganization in the sensorimotor cortex, thereby potentially contributing to pain relief.

Chronic pain is often accompanied by negative emotions such as anxiety and depression. However, most patients in our study presented with normal psychological status, and only a small proportion had mild anxiety and depression (cutoff score = 50). The total symptom index of SCL-90 ranged from asymptomatic to mild distress (cutoff value = 1.5). Correlation analysis revealed no significant association between pain scores and psychological questionnaire scores, except that higher anxiety and depression levels were accompanied by elevated SCL-90 scores. These results may be influenced by individual differences, social and family environments, as well as the relatively short follow-up duration. No statistically significant psychological changes were observed in the current sample during the observed follow-up period, given the relatively mild baseline psychological burden among recruited patients and the small sample size, which was insufficient to comprehensively evaluate psychiatric outcomes. Notably, MVF therapy introduced heterogeneous effects on patients’ sleep status. Improved sleep was observed in several participants, possibly due to pain alleviation, whereas a few individuals experienced sleep disturbance, which may be linked to individual tolerance to the intervention. Such divergent responses reflect the complex influence of MVF on sleep and deserve more targeted evaluation in future studies. Moreover, some patients exhibited improved relaxation and motor function of the affected limb during MVF therapy, indicating that MVF therapy may support early sensorimotor recovery in patients with TBPI after surgery, which merits in-depth exploration.

After 6 weeks of MVF therapy, Patient 3 showed no pain relief, with frequent training interruptions due to severe pain and impaired concentration. Following dorsal root entry zone (DREZ) lesioning, the patient’s pain was completely resolved, with only mild weakness of the affected lower limb remaining, and high satisfaction with the outcome. This case indicates that DREZ lesioning may represent a potential alternative for refractory neuropathic pain following responsiveness to MVF therapy, despite its inherent risks and potential complications, warranting further investigation. Notably, all patients enrolled in our study underwent brachial plexus nerve transfer or neurolysis surgery and were included only at more than 1 month postoperatively. The specific inclusion and exclusion criteria were as follows. First, patients with acute pain caused by fresh TBPI were excluded, as such pain usually resolves gradually over time with surgical repair and pharmacological intervention. Second, TBPI patients often present with severe functional impairment and generally undergo early surgical intervention under current clinical guidelines; very few patients decline surgery, which is consistent with the overall clinical profile of the study population. This study focused specifically on early-onset and persistent neuropathic pain following TBPI surgery, a type of pain rarely observed in patients with intact nerve trunks or in the preoperative setting. Recruiting postoperative patients effectively excluded confounding effects from peripheral mechanisms such as neuroma formation, helping facilitate investigation into the central mechanisms underlying intractable pain. The inclusion cutoff of ≥1 month postoperatively eliminated interference from acute postoperative pain. Despite objective variations in TBPI severity and surgical procedures among patients, this study concentrated on intractable pain persisting after nerve neurolysis or transfer, aiming to clarify the possible pivotal role of central mechanisms in the development and progression of intractable pain in patients with poor functional recovery following nerve injury.

### 4.5 Limitations

This study has several notable limitations. First, due to the COVID-19 pandemic and the study’s duration, the sample size was relatively small. Additionally, there was participant attrition throughout the research, which could potentially weaken the statistical reliability of our analyses. Second, subgroup analyses stratified by lesion laterality resulted in even smaller subsamples, which may reduce statistical power and limit the reliability and generalizability of laterality-related findings; all subgroup results should therefore be regarded as exploratory. Third, some participants were unable to perform actual movement due to motor dysfunction and instead completed only the motor imagery component of the intervention. While motor imagery shares some neural processing pathways with actual movement, the two conditions involve distinct neural mechanisms, which may have introduced heterogeneity in the intervention effects across participants. Collectively, these methodological confounders highlight that the long-term efficacy of MVF therapy still requires further validation in larger-sample prospective studies; ideally, future trials should incorporate prespecified subgroup analyses stratified by baseline motor function to adjust for interindividual intervention heterogeneity.

## 5 Conclusion

The results from this study indicate that MVF therapy may help relieve neuropathic pain following TBPI. MVF therapy is easy to implement, involving only basic grasping, picking, and sensory training. It features simple equipment requirements, low resource requirements, low cost, and suitability for home-based practice, with mild adverse reactions. Our preliminary findings suggest that the implementation of MVF therapy in treating neuropathic pain after TBPI may be a potentially viable intervention, and more investigations for clinical applications are warranted.

## Data Availability

The original contributions presented in the study are included in the article/Supplementary material, further inquiries can be directed to the corresponding author.
