# Supplementary material for: Therapeutic effect of mirror visual feedback therapy on neuropathic pain in patients with total brachial plexus injuries
Source: Front Neurol. 2026 Aug 5;17:1875550. doi: 10.3389/fneur.2026.1875550 (PMC13487496; doi:10.3389/fneur.2026.1875550)
Supplement: Supplementary file 1 [file Data_sheet_1.DOCX]

Supplementary Material

# 1 Supplementary Figures and Tables

## 1.1 Supplementary Figures

**Supplementary Figure 1.** Group-level axial brain activation maps during hand motor tasks in the left TBPI group. Top, middle, and bottom rows represent left-hand, right-hand, and bimanual motor tasks, respectively. Left and right columns indicate pre-treatment and post-treatment activation, respectively.

**Supplementary Figure 2.** Group-level axial brain activation maps during hand motor tasks in the right TBPI group. Top, middle, and bottom rows represent left-hand, right-hand, and bimanual motor tasks, respectively. Left and right columns indicate pre-treatment and post-treatment activation, respectively.

**Supplementary Figure 3.** Group-level axial brain activation maps during hand motor tasks in the healthy control group. Top-left panel: left-hand motor task; top-right panel: right-hand motor task; bottom panel: bimanual motor task.

## 1.2 Supplementary Tables

**Supplementary Table 1.** Baseline characteristics of TBPI patients with neuropathic pain.

| **Patient No.** | **Age (years)** | **Gender** | **Affected side** | **Time to first fMRI post-injury (months)** | **Cortical reorganization** |
| --- | --- | --- | --- | --- | --- |
| 1 | 34 | Male | Left | 30 | Touching the affected cheek and ear root has the illusion that the forearm is touched on the flexion side |
| 2 | 21 | Male | Left | 5 | None |
| 3 | 46 | Male | Left | 30 | None |
| 4 | 26 | Male | Left | 4 | None |
| 5 | 16 | Male | Left | 14 | None |
| 6 | 53 | Male | Left | 60 | None |
| 7 | 22 | Male | Left | 1 | None |
| 8 | 25 | Female | Left | 24 | Touching the affected side of the patient's face has the sensation of touching the affected wrist and forearm |
| 9 | 51 | Male | Left | 252 | None |
| 10 | 45 | Male | Left | 60 | None |
| 11 | 33 | Male | Right | 10 | Touching the lateral side of the upper arm can have a discharge sensation to the hand, while sometimes there is a spontaneous sensation of phantom limb activity |
| 12 | 24 | Male | Right | 4 | None |
| 13 | 18 | Male | Right | 3 | None |
| 14 | 16 | Male | Right | 16 | Touching the affected cheek has the sensation of being touched on the flexed side of the forearm |
| 15 | 31 | Male | Right | 108 | Numbness in the palms of the affected limbs when touching the cheeks and jaws bilaterally, and illusion of motor activity of the affected limbs during daily motor tasks involving the healthy limbs |
| 16 | 20 | Male | Right | 60 | None |
| 17 | 22 | Male | Right | 48 | None |
| 18 | 24 | Male | Right | 30 | Touching the affected side of the jaw and the feeling of touching the affected hand, and the feeling of muscle contraction of the affected hand from the foot activity |

**Supplementary Table 2.** Comparison of baseline characteristics between TBPI patients with neuropathic pain and healthy controls.

| **Variables** | **Patient (n=18)** | **Control (n=25)** | ***P*** |
| --- | --- | --- | --- |
| Age (years) | 29.28±11.30 | 29.04±4.94 | 0.929^a^ |
| Female, n (%) | 1 (5.6) | 2 (8.0) | 0.059^b^ |

^a^ Independent-samples *t*-test (two-tailed); ^b^ Chi-square test.

**Supplementary Table 3.** Baseline pain characteristics in TBPI patients with neuropathic pain.

| **Patient No.** | **VAS score** | **Nature of pain** | **Pain frequency** | **Sleep impact** |
| --- | --- | --- | --- | --- |
| 1 | 5 | Distending pain | Paroxysmal | Normal |
| 2 | 5 | Pricking | Paroxysmal | Normal |
| 3 | 9 | Dragging pain | The pain is severe, with the head tilted to the affected side while the healthy hand clutches the affected limb, and lasts for more than 10 seconds before relieving | Normal |
| 4 | 4 | Chirobrachialgia | Persistence | Affect sleep |
| 5 | 9 | Electric pain | Paroxysmal, occurring every 10 minutes | Normal |
| 6 | 5.5 | Distending pain and constriction of the affected limb | Persistent attacks that require massage of the arm muscles to relieve each time. Cold sensitivity | Normal |
| 7 | 5 | Pricking pain | Paroxysmal | Normal |
| 8 | 6.5 | Electric pain | Paroxysmal, pronounced at night | Affect sleep |
| 9 | 5.5 | Electric pain | Persistent, pain worsens when the affected limb is cold or after drinking alcohol | Difficulty in falling asleep |
| 10 | 6.5 | Chirobrachialgia | Postoperative aggravation, multiple times a day, lasting 10 minutes or so each time | Normal |
| 11 | 6 | Burning pain | Paroxysmal | Normal |
| 12 | 7 | Chirobrachialgia | Paroxysmal, more than 20 times a day | Waking up at night due to numbness and poor sleep quality |
| 13 | 4.5 | Electric and pricking pain | Paroxysmal, about 3 times a week | Normal |
| 14 | 4 | Chirobrachialgia | Paroxysmal, irregular | Normal |
| 15 | 7 | Distending pain | Persistent attacks, 1-2 times a week with particularly intense pain, cold sensitivity | Affect sleep |
| 16 | 6 | Dragging pain | Persistent, stopping occasionally, light and heavy at times | Normal |
| 17 | 4 | Squeezing pain | About twice a day | Affect sleep |
| 18 | 5.5 | Gnawing pain | Irregular pain during the day, fixed pain once before bedtime, last a few dozen seconds to more than a minute | Normal |
